# Positional Information Generated by Spatially Distributed Signaling Cascades

**DOI:** 10.1371/journal.pcbi.1000330

**Published:** 2009-03-20

**Authors:** Javier Muñoz-García, Zoltan Neufeld, Boris N. Kholodenko

**Affiliations:** 1 School of Mathematical Sciences and Complex Adaptive Systems Laboratory, University College Dublin, Dublin, Ireland; 2 Grupo Interdisciplinar de Sistemas Complejos (GISC), Madrid, Spain; 3 UCD Conway Institute, University College Dublin, Dublin, Ireland; 4 Department of Pathology, Anatomy and Cell Biology, Thomas Jefferson University, Philadelphia, Pennsylvania, United States of America; University of Washington, United States of America

## Abstract

The temporal and stationary behavior of protein modification cascades has been extensively studied, yet little is known about the spatial aspects of signal propagation. We have previously shown that the spatial separation of opposing enzymes, such as a kinase and a phosphatase, creates signaling activity gradients. Here we show under what conditions signals stall in the space or robustly propagate through spatially distributed signaling cascades. Robust signal propagation results in activity gradients with long plateaus, which abruptly decay at successive spatial locations. We derive an approximate analytical solution that relates the maximal amplitude and propagation length of each activation profile with the cascade level, protein diffusivity, and the ratio of the opposing enzyme activities. The control of the spatial signal propagation appears to be very different from the control of transient temporal responses for spatially homogenous cascades. For spatially distributed cascades where activating and deactivating enzymes operate far from saturation, the ratio of the opposing enzyme activities is shown to be a key parameter controlling signal propagation. The signaling gradients characteristic for robust signal propagation exemplify a pattern formation mechanism that generates precise spatial guidance for multiple cellular processes and conveys information about the cell size to the nucleus.

## Author Summary

Living cells detect environmental cues and propagate signals into the cell interior employing signaling cascades of protein modification cycles. A cycle consists of a pair of opposing enzymes controlling the activation and deactivation of a protein, where the active form transmits the signal to the next cascade level. A crucial challenge in cell and developmental biology is to understand how these cascades convey signals over large distances and how spatial information is encoded in these signals. With the advent of advanced imaging techniques, there has been emerging interest in understanding signal propagation in cells and tissues. Based on a simple cascade model, we determine the conditions for signal propagation and show how propagating signals generate spatial patterns that can provide positional information for various cellular processes.

## Introduction

Cascades of covalent protein modification cycles convey signals from cell-surface receptors to target genes in the nucleus. Each cycle consists of two or more interconvertible protein forms, for example, a phosphorylated and unphosphorylated protein, and an active, phosphorylated protein signals down the cascade. In eukaryotes, post-translational protein modifications include phosphorylation of Tyr, Thr and Ser residues, ubiquitylation, acetylation or sumoylation of Lys, methylation of Arg and Lys, and other modifications [1]. Every protein modification cycle is catalyzed by two opposing enzymes, such as a kinase and phosphatase for (de)phosphorylation cycle, ubiquitin ligase and deubiquitylating isopeptidase for (de)ubiquitylation, and methyltransferase and amine oxidase demethylase for (de)methylation. Well known examples of signaling cascades include mitogen activated protein kinase (MAPK) cascades, small GTPase cascades and coagulation cascades in blood clotting [2]–[4]. Instructively, although a MAPK cascade is usually referred to as a 3-tier pathway, in fact, the cascade encompasses five or more layers, which sequentially activate each other [5].

While signaling cascades were studied experimentally and theoretically for more than half a century, most studies disregarded the spatial aspects of signal propagation, considering one or more well-mixed compartment(s) with no variation in spatial dimensions. The stationary and temporal behavior of protein modification cascades was extensively analyzed, starting from pioneering numerical simulations by Stadtman and Chock [6] and followed by a theoretical exploration of steady-state input-output responses for a signaling cycle by Goldbeter and Koshland, who coined the term ultrasensitivity [7]. Depending on the degree of saturation of opposing enzymes in a modification cycle, the response curve for either interconvertible form varies from a merely hyperbolic to an extremely steep sigmoidal function [7]. Subsequent work showed that an increase in the number of layers in a cascade can further increase the sensitivity of the target to the input signal [8],[9]. Although the dynamics of temporal responses of signaling cascades to a sustained, decaying or pulse-chase stimulation received less attention, the major types of temporal responses have been described. Depending on the cascade architecture and kinetic parameters, the sustained input can evoke sustained, transient [10],[11] or more complex, bistable [12],[13] and oscillatory responses [14]–[16]. An exponentially decaying input, which approximates the activity of a receptor after stimulation by a step function, causes a transient cascade response [17]. Yet, despite important breakthroughs in understanding the input-output relationships and temporal dynamics of information processing, we currently lack sufficient theoretical and experimental insights into spatial propagation of signals generated by protein modification cascades [18],[19].

External signals received at the plasma membrane have to propagate across the cell to reach their targets, and, therefore, protein diffusion and active transport can change quantitative and qualitative aspects of output signaling by protein cascades [20]–[26]. In fact, signaling cascades are spatially distributed in living cells. Often, activating signals are only present on the cell membrane where activated receptors and small G-proteins (such as Ras and Rap that activate MAPK cascades) reside, whereas inactivating signals (carried out by phosphatases in MAPK cascades) are distributed throughout the cytoplasm. The concept of protein activity gradients that arise from the spatial separation of opposing enzymes in a protein modification cycle was proposed fairly recently [27]. For a protein phosphorylated by a membrane-bound kinase and dephosphorylated by a cytosolic phosphatase, Brown & Kholodenko predicted that there can be a gradient of the phosphorylated form, with high concentration close to the membrane and low within the cell [27]. This prediction was confirmed after a few years, when biosensors based on fluorescence resonance energy transfer enabled the discovery of activity gradients of the small GTPase Ran [28], microtubule-binding protein stathmin [29], the yeast MAPK Fus3 [30], and, very recently, the anaphase phosphorylation gradient of Aurora B kinase, which, as a part of the chromosome passenger complex controls microtubule attachments to kinetochores and the late stages of cell division [31].

Precipitous gradients of phosphorylated kinases can impede information transfer from the plasma membrane to distant cellular locations, such as the nucleus. In the Ras/Raf/Mek/ERK (MAPK) cascade, Ras, Raf and, partially, MEK activation is localized to the plasma membrane, whereas MEK and ERK deactivation by phosphatases occurs in the cytoplasm. Calculations [32],[33] and the experimental data [30] suggest that gradients of phosphorylated MEK and ERK can be steep, when the phosphorylated signals are terminated by phosphatases at the distances of 3–5 µm and greater from the plasma membrane. Despite recent work on the long-range signal transfer by phosphoprotein waves [33], the quantitative understanding of how activity gradients spread in the space by the subsequent levels of signaling cascades is still lacking. It is not understood how the spreading of phosphorylation signals depends on the number of cascade levels (stages) and how the gradients of phosphoproteins along the cascade are controlled by the kinetic properties of the kinases and phosphatases. The previous work suggested that having more layers in a cascade would spread the phosphorylation signal to span ever increasing distances from the activation source [14],[27]. The present paper shows that this simplistic view should be improved, and if the ratio of the phosphatase and kinase activities is above a certain threshold, the propagation of the phosphorylation signal stalls in the space. We demonstrate that a signaling cascade can produce a set of steady-state activation profiles that precipitously decay at different locations for different levels of the cascade. We determine analytically these locations, relating them with the kinetics of component reactions in signaling cascades. These activation profiles provide the localization information, which then can be used by other signaling pathways to regulate a range of cellular processes. We determine the features and conditions of the signal propagation and investigate the effect of saturation of activation and deactivation reactions on the generation of positional information.

## Model

### Model of a spatially distributed signaling cascade

**Figure 1:**
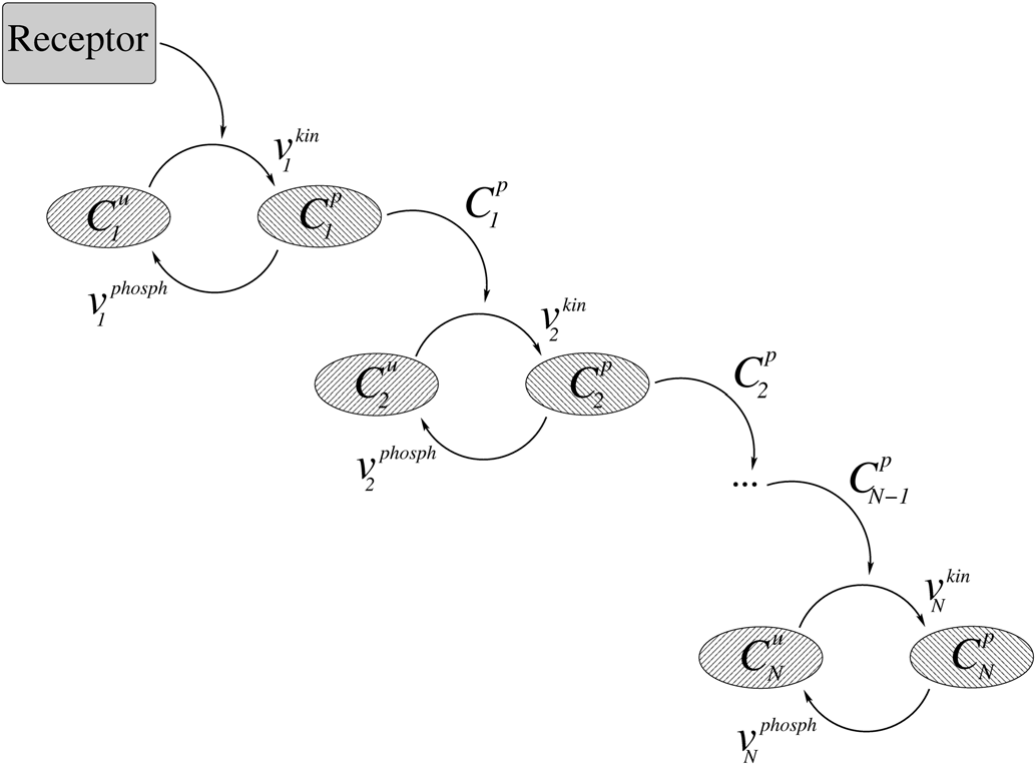
Cascade signaling scheme.

Spatial separation of opposing enzymes, such as kinase and phosphatase, are hallmarks of protein modification cascades, including MAPK cascades. Here we consider a cascade of protein modification cycles, where each cycle consists of inactive and active forms of a signaling protein, and the active form catalyzes the activation of the protein at the next level down the cascade (Fig. 1). Although our analysis applies to any kind of protein modification, for convenience we will use the terminology of protein phosphorylation and dephosphorylation and a cascade of protein kinases as an example. The initial diffusible kinase in the cascade is activated exclusively at the plasma membrane by the membrane-bound receptor or small GTPase, whereas downstream kinases are phosphorylated in the cytoplasm by an active, diffusible kinase of the upstream level. A phosphorylated kinase is dephosphorylated in the cytoplasm by an opposing phosphatase at each cascade level.

A simplified model, which neglects protein sequestration effects [34],[35], describes the signaling system in terms of the concentrations of the phosphorylated protein, 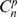, and the concentration of its unphosphorylated form, 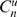, at each level *n* of the cascade. The spatio-temporal dynamics of the phosphorylated kinases are governed by the following reaction-diffusion equations:
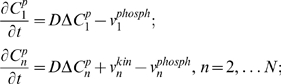
(1)where *D* is the diffusion constant, and 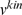 and 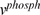 are the phosphorylation and dephosphorylation rates, catalyzed by kinases and phosphatases, respectively. The equation for 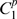 lacks the phosphorylation rate term, as the first level kinase is phosphorylated solely at the membrane.

Spherical symmetry simplifies analysis of signaling in three dimensions, as the protein concentrations become functions of the radial distance and time only [27]. For simplicity, we neglect curvature effects and further consider a one-dimensional reaction-diffusion system with the Cartesian spatial coordinate *x* and the first kinase activated at the pole *x* = 0 (the plasma membrane). For this kinase, the diffusive flux at the membrane (*x* = 0) equals the surface phosphorylation rate, 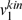, and zero at the opposite pole, *x* = *L*. For kinases at the subsequent levels, there is no diffusive flux at either pole, which gives the following boundary conditions for Eq. (1):
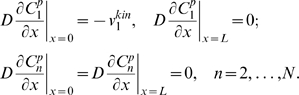
(2)


When diffusivities (*D*) of the phosphorylated and unphosphorylated forms are equal, and *de novo* protein synthesis and degradation are negligible on the time scale considered, the total protein abundance at each cascade level, 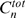, is constant across the cell [19],
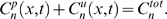
(3)


Assuming that the kinases and phosphatases follow Michaelis-Menten kinetics, at each level the phosphatase rate depends on the phosphorylated form concentration, 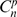, whereas the kinase rate depends on the concentration of the unphosphorylated protein, 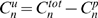, and the concentration of the active kinase, 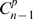, at the immediately preceding level,
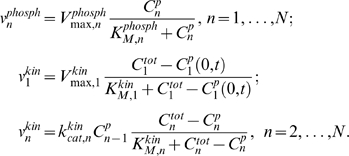
(4)Here 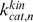, 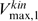, and 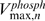 are the catalytic constant (turnover number) and the maximal rates. 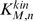 and 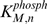 are the Michaelis constants of the kinase and phosphatase at the *n*-th level [14]. Note that, in contrast with downstream levels, the surface rates 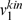 and 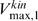 have the same dimension as the diffusion flux (e.g, m·sec^−1^).

It is convenient to use the normalized protein concentrations 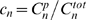. Dividing Eqs. (1)–(4) by 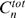, and bearing in mind that the unphosphorylated fractions are given by 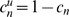, we simplify the description of the cascade dynamics as follows
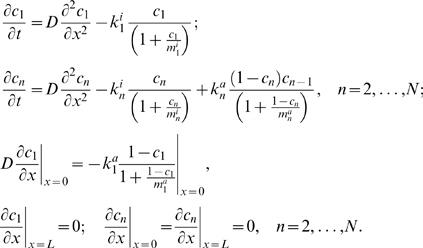
(5)Here 
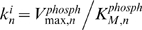
 are the phosphatase activities (known as the apparent first-order rate constants for a linear kinetic domain), 
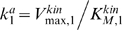
 and 
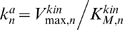
 for *n* = 2, …, *N*, are the kinase activities, where the maximal kinase rate is 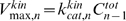 for *n* = 2, …, *N*. The dimensionless (normalized) Michaelis constants of the phosphatases and kinases are 
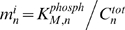
 and 
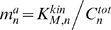
, respectively. The enzyme activities 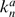 and 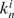 determine the temporal scale of chemical reactions, indicating how fast phosphorylation and dephosphorylation occur, whereas the normalized Michaelis constants 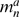 and 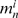 characterize how far these reactions are from saturation (unsaturated reactions correspond to 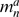, 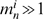).

## Results

### Signaling cascades where reactions are far from saturation

In this section we study the case when the total concentrations are small compared to the Michaelis constants, or in terms of the non-dimensional parameters, 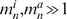. From Eq. (5), it follows that the evolution of the concentration of the active component at the first level is given by
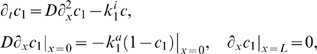
(6)where we used the notation, 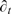 and 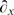, for the partial derivatives with respect to time *t* and the spatial coordinate *x*. The stationary solution to Eq. (6) reads,
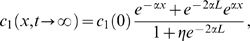
(7)where 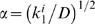, 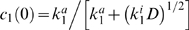, and 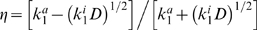. The distance 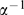 defines the characteristic length scale for the gradient of the first kinase activity. When the spatial domain is large, that is 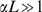, the activation profile of the first kinase decays almost exponentially, 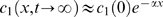. Note, that regardless of the particular kinetics of the activation at the membrane, the steady-state profile 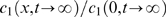 of the first level kinase always decays nearly exponentially for large domains [27]. Using typical values of *D* = 5 µm^2^ s^−1^, 
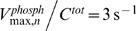
 and 
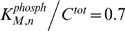
 as in Ref. [14], the characteristic length scale can be estimated as 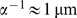.

In addition to solving Eq. (5) numerically, we will explore analytically how the kinase activation profiles spread from the cell membrane into the cell interior. To simplify the analysis, we will further assume that the phosphatase activities 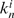 for *n* = 1, …, *N* and the kinase activities 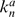 for *n* = 2, …, *N* do not vary over different cascade levels (although the values of the maximal rates, Michaelis constants and the total protein concentrations can be unique for each individual level). Non-dimensionalizing Eq. (5) by using the characteristic length scale 1/α, as 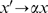, and the temporal scale 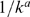, 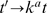, we obtain (omitting primes)
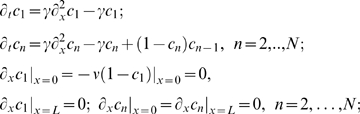
(8)where 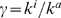 is a key parameter equal to the ratio of the phosphatase and kinase activities (the ratio of deactivation and activation rates for a general case). The parameter 
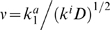
 indicates the strength of the membrane signal that determines the phosphorylation level of the first kinase at the membrane, as 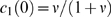.

**Figure 2:**
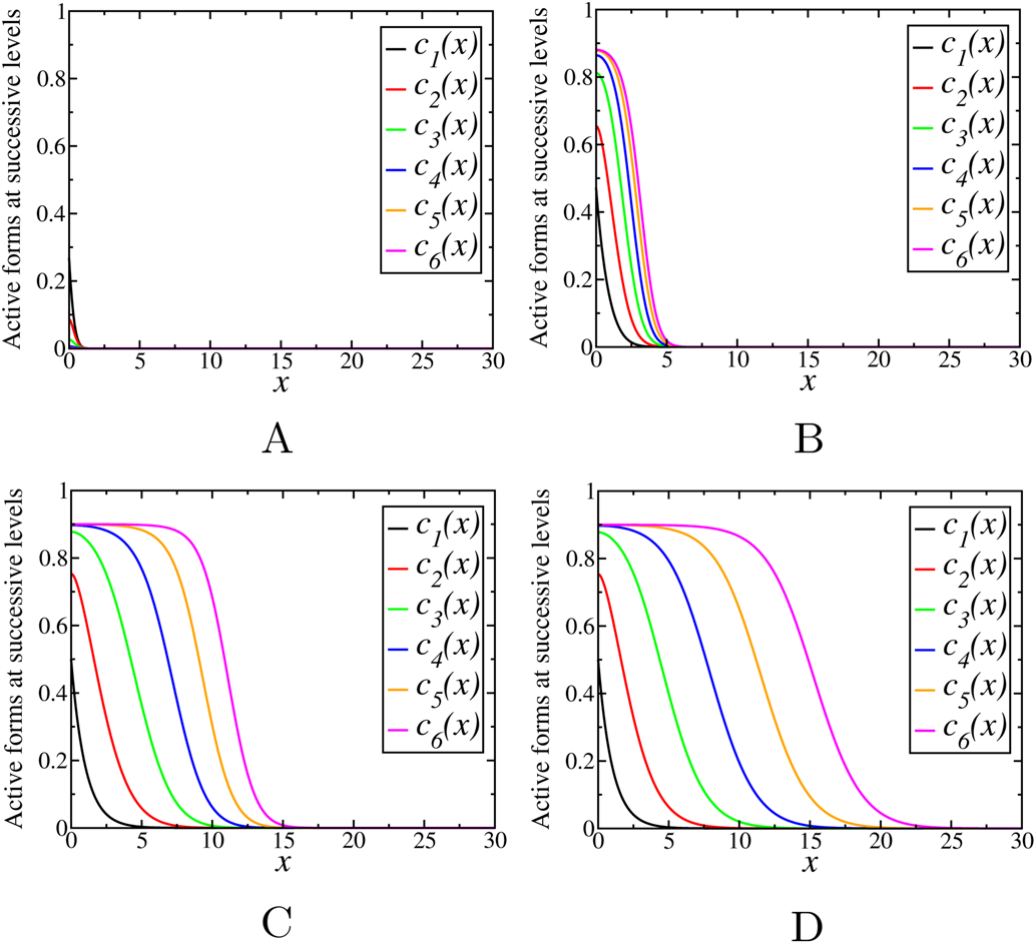
Active form concentration profiles, 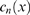, obtained by numerical integration of Eq. (8) with γ = 0.1 at different times: (A) *t* = 1; (B) *t* = 10; (C) *t* = 50; (D) 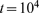.

We will first examine numerical solutions of Eq. (8) with the initial conditions 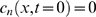, *n* = 1,…,*N* on a domain of size *L* = 100 and 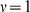. These numerical solutions are shown in Fig. 2 for six consecutive cascade levels. Note that the initial conditions and the signal strength 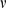 do not affect the qualitative behavior of solutions (see the supporting information, Figs. S1 and S2). For γ = 0.1, the concentration profiles propagate into the domain, moving to the right, until stationary profiles are attained (Fig. 2D). The stationary concentration profiles propagate more deeply into the spatial domain as *n* increases, i.e. at higher levels of the cascade. A similar behavior is found for other values of γ<1.

For γ>1 we find a different scenario (see Fig. S3). In this case, the concentration profiles remain localized to the region near the left boundary, and their amplitudes decay dramatically for the consecutive levels. Thus, in this case the signal does not propagate through the domain and does not reach the right boundary.

**Figure 3:**
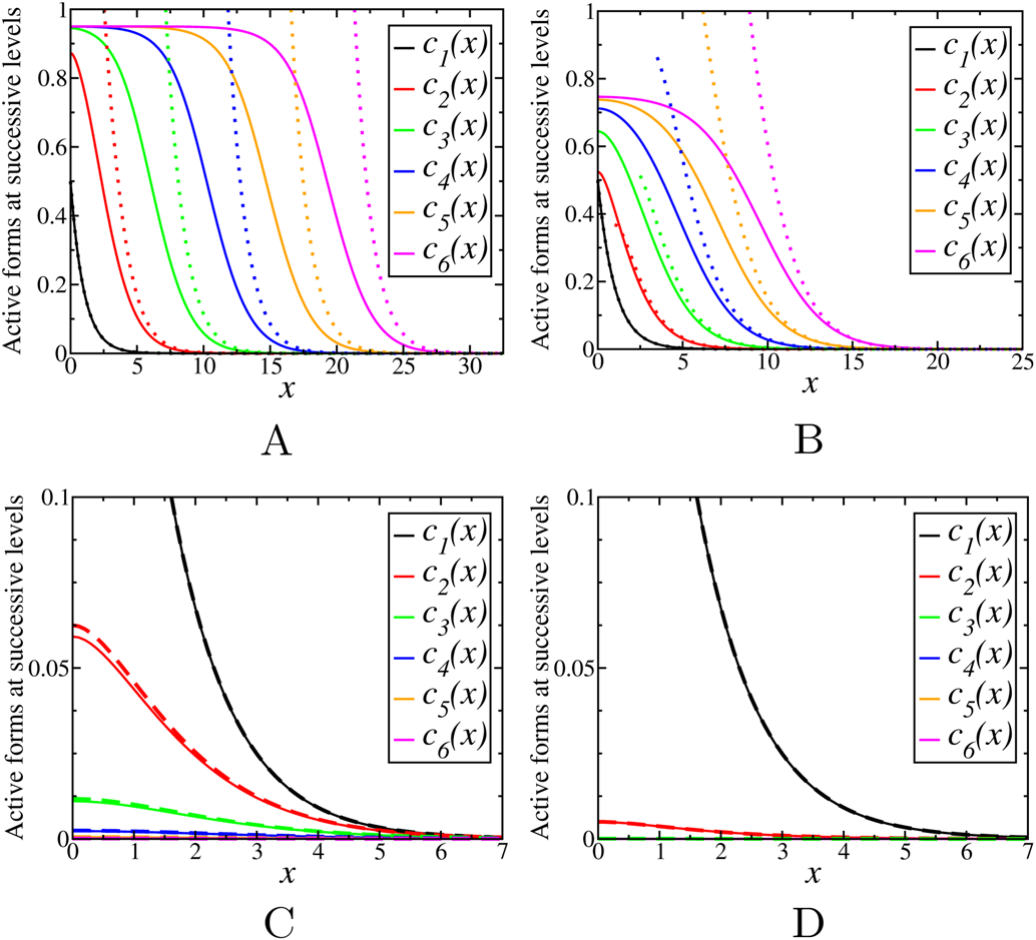
Stationary concentration profiles, 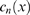, for different values of γ: (A) γ = 0.05; (B) γ = 0.25; (C) γ = 4; (D) γ = 50. The dotted lines in Fig. (A) and (B) are given by Eq. (10). Dashed lines in Figs. (C) and (D) are the exact solutions to Eq. (9) given in the supporting information by Eq. (A2).

Examples of the asymptotic, steady state solutions are shown in Fig. 3 for a range of the ratios γ of the phosphatase and kinase activities. When γ<1, the width of the profiles increases for smaller γ, while for γ>1 larger values of γ result in a faster decay with the concentration profiles localized to the left boundary. Note that the stationary solution for the first level is independent of γ.

In general, there is no simple analytical expression for the stationary solutions of Eq. (8), but we can gain some insight considering the behavior of these solutions within a range where 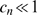. This approximation is always valid for the tail of the spatial distribution, that is for sufficiently large *x*, but when γ>1, it can be satisfied over the whole domain. In this case, we can rewrite Eq. (8) in the stationary state as
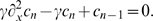
(9)This is a system of second order linear differential equations, which can be solved successively to obtain 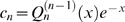 where 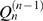 is a polynomial of order 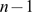 (explicit expressions are given in Text S1). Figs. 3C and 3D (dashed lines) illustrate that for γ>1 the analytical solution of Eq. (9) agrees well with the numerical results.

**Figure 4:**
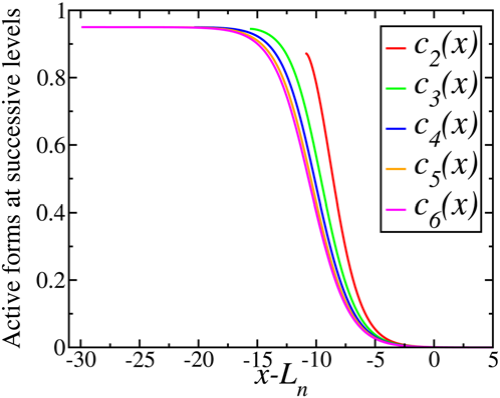
Concentration profiles as in Fig. 3A shifted to the left by a distance 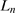 given by Eq. (11) with 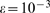.

Importantly, for γ substantially less than 1, we can determine the propagation length for successive activation profiles by using Eq. (9) near the tail of the distribution. Since the approximation 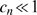 is not valid near the boundary *x* = 0, the coefficients of the polynomial 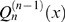 cannot be exactly calculated (Text S1). However for small values of γ, when the activation signal spreads far enough 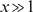 for the tail region, the dominant contribution to the solution comes from the largest order term in 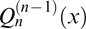 which can be exactly obtained (see Text S1),
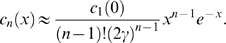
(10)These solutions are shown (dotted lines) in Fig. 3A and B for different values of γ. Thus Eq. (10) gives a simple analytical approximation to describe the tail of each stationary profile. We will use this approximation to obtain the propagation length of the activation profile at different levels of the cascade. We formally define the propagation length of the steady state solution, 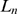, as the coordinate 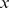 where the concentration falls below a certain threshold 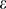. Using Eq. (10) we have 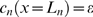, which gives an implicit equation for the length, 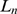, that can be expressed by the Lambert *W* function [36] (or simply solved numerically),
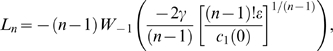
(11)where the index −1 denotes the solution branch of the Lambert function corresponding to the values of 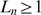. In the original dimensional units this length corresponds to 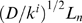, hence the spatial spread of signaling by each phosphorylated kinase is well characterized by the propagation length *L_n_*. Figure 4 shows that if the concentration profiles for the successive cascade levels are shifted to the left by the distance 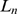 given by Eq. (11) with 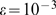, the resulting profiles 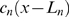 converge to a single curve. Thus, Fig. 4 demonstrates that the steady state profiles have flat plateaus, which start from the left boundary. The length of each plateau, where the concentration is almost constant, increases with the cascade level, *n*. The plateau is followed by a transition region where the concentration decreases to zero. The shape of the curve corresponding to the transition region is asymptotically independent of *n*, with faster convergence to this asymptotic form for smaller values of γ.

We can also determine the maximum active concentration (termed the maximal signaling amplitude) 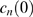 for 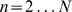 that corresponds to the plateau region. Since the concentration field in the plateau region is flat, the second derivative can be neglected. Assuming that *n* is sufficiently large, 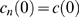 is independent of *n*, and using Eq. (8), we obtain,
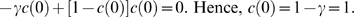
(12)Note that a positive solution for 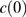 only exists for γ<1, that is consistent with the observation that the signal cannot propagate into the spatial domain when γ>1.

Assuming the same profile shape for the different levels, we can use Eqs. (11) and (12) to estimate the total amount of active component 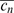 for small values of γ. The 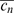 value presents the stationary cascade activation response integrated over the space. At each level this spatial integral response should be proportional to the propagation length of the signal, 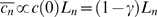. The asymptotic expansion of *L_n_* is proportional to 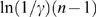, thus the total active component of the different cascade levels depends on γ according to the functional form 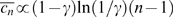. This indicates that the difference in the total activated concentration at consecutive levels is 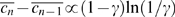, hence there is a constant step size between consecutive levels proportional to 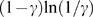 which agrees with the numerical results shown in Fig. S4.

### Signaling cascades with saturable enzymes

In this section we consider a more general case given by Eqs. (5) that allows for saturation kinetics. Following the rescaling as in the previous section, the dynamics of the concentrations is described as follows,
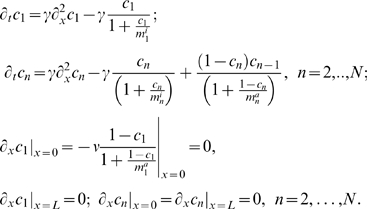
(13)For large values of 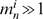 and 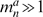, Eq. (13) reduces to Eq. (8).

As in the previous section, we consider a cascade with similar properties for all levels and assume 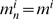, 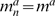. Note, that in contrast with the unsaturated case, we now make an additional assumption that the degrees of saturation of kinases and phosphatases do not depend on the cascade level, which implies that the corresponding Michaelis constants should change nearly proportionally to the total protein concentrations at each level.

**Figure 5:**
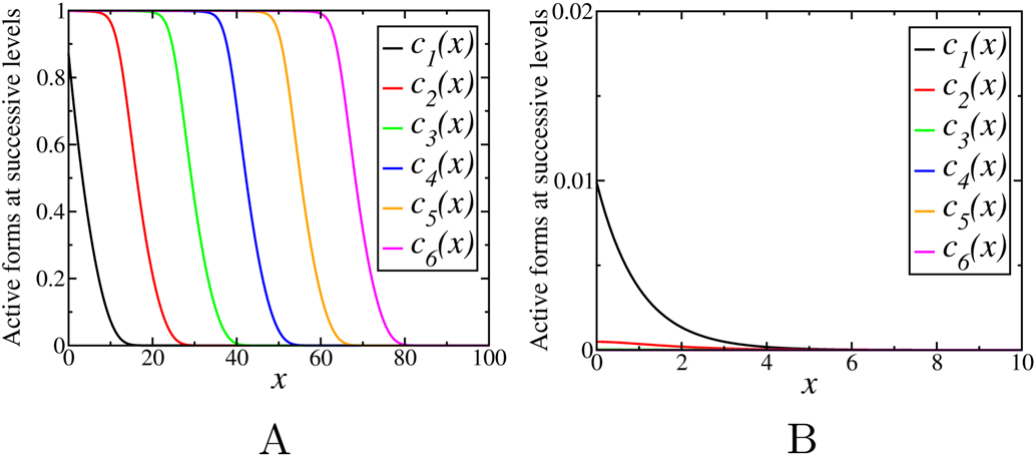
Stationary concentration profiles, 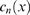, obtained by numerical integration of Eqs. (13) with γ = 0.1 for different values of 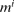 and 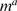: (A) 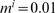, 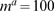; (B) 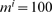, 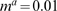.

**Figure 6:**
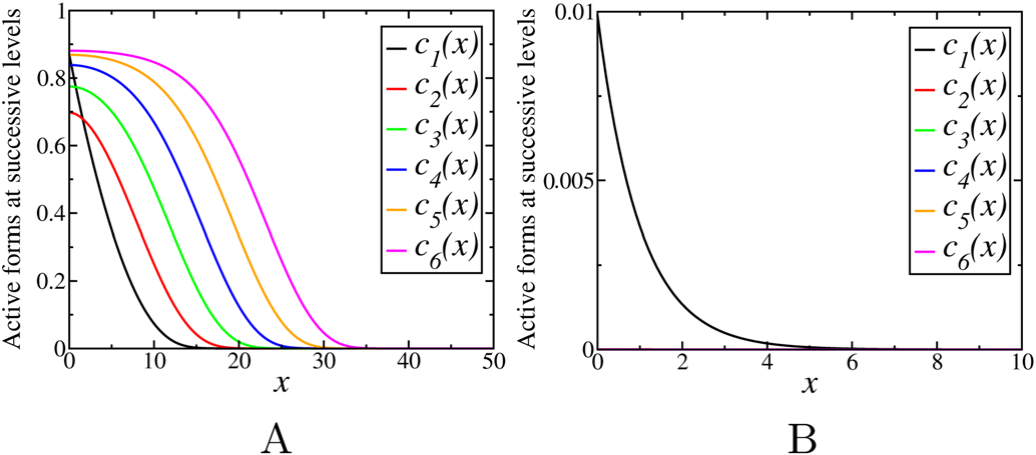
Stationary concentration profiles, 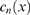, obtained by numerical integration of Eqs. (13) with *γ* = 10 for different values of 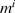 and 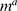: (A) 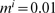, 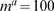; (B) 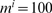, 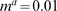.

Simulations show that for saturable kinetics, the final steady states are not affected by the initial conditions, similarly as above (Fig. S5). Although the behavior of the phosphorylation profiles is found to be qualitatively similar to the case of non-saturable kinetics, it depends not only on the phosphatase/kinase activity ratios (γ), but also on the degree of saturation. For fixed values of *m^i^* and *m^a^*, depending on the ratio γ, the stationary concentration profiles down the cascade either decay with *n*, or propagate into the spatial domain covering increasing distance with an increase in *n*. However, in contrast to the previous section where 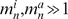, the threshold value separating these two different behaviors is different from unity and depends on the Michaelis constants 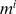 and 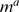. Figs. 5 and 6 show examples of the steady state activation profiles for γ = 0.1 and γ = 10 for different values of 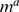 and 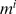. Note, that when deactivation reactions saturate at low values 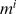, whereas activation reactions have higher values 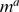, the signal may propagate even for γ greater than 1, whereas it can decay for γ much less than 1, for the opposite relation between the Michaelis constants.

**Figure 7:**
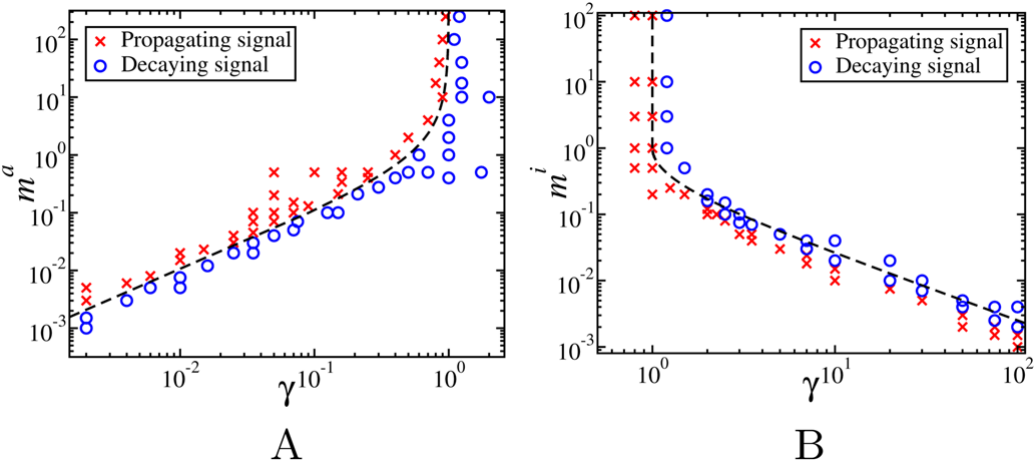
Parameter regions corresponding to propagating (red crosses) and decaying signals (blue circles) as a function of γ and 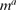 or 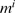 for: (A) 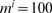, and (B) 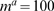. Dashed lines in Fig A and B are given by the Eqs. (16) and (18), respectively.

To illustrate the threshold behavior of the signal propagation in the parameter space, we consider two cases of large 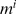 and large 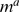 separately, each corresponding to systems where one of the opposing reactions is far from saturation. Fig. 7 shows phase diagrams of the system indicating the boundary between the decaying and propagating signals on the γ, 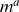 plane for a fixed value of 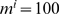, and on the γ, 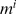 plane for 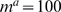, respectively.

For 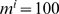, Fig. 7A shows that for large values of 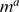, the threshold between propagating and decaying signals is γ = 1 in agreement with the linear case discussed in the previous section, but for 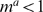, much lower values of γ are necessary for the propagation. On the other hand, if 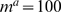 (Fig. 7B), we obtain propagation for all γ<1, however the saturation of the inactivating reaction extends the threshold to larger values of γ when 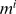 is small.

To obtain an analytical approximation for the boundary between the two regimes of decaying or propagating signals, as in the previous section, we assume that a propagating signal produces a set of stationary concentration profiles with a flat plateau region on the left side of the domain and the concentration 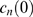 converges to a constant for large *n*. The existence of non-zero asymptotic concentration, 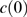, is a prerequisite for the efficient signal propagation. Neglecting the second derivative at *x* = 0 from Eq. (13) we obtain
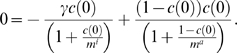
(14)


We will first assume that the phosphatases are far from saturation, 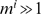, whereas the kinases can be saturable. Since 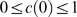, we can simplify Eq. (14) to obtain
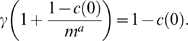
(15)We can readily see that Eq. (15) has a solution within 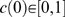 only if
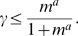
(16)When the ratio γ of opposing enzyme activities satisfies Eq. (16), the solution of Eq. (15) is 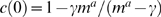. The dashed line in Fig. 7A represents the curve 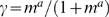 given by Eq. (16), that agrees well with the numerical results.

When the kinases in the cascade are far from saturation, 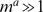, from Eq. (14) we obtain the following equation for 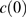,
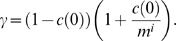
(17)The right hand side of Eq. (17) is a parabola with a maximum at 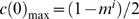 where its value is 
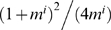
. However, when 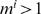, the maximum is reached for negative concentration values, and in this case the maximum for positive concentrations is 1. Thus, Eq. (17) only has a positive solution within the interval [0,1] if
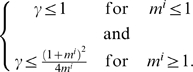
(18)The dashed lines in Fig. 7B represent the curves 
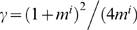
 and *γ* = 1 as given by (18).

**Figure 8:**
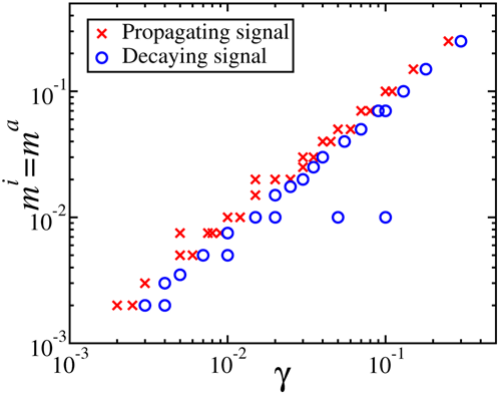
Parameter regions corresponding to propagating (red crosses) and decaying signals (blue circles) as a function of γ and small values of 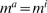.

Another regime of qualitatively different behavior is the case when both reactions are saturated, i.e. 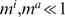. The condition for signal propagation in this case cannot be obtained analytically from the plateau solution of Eq. (14) as in the previous cases, since this only gives an approximation for an unstable solution 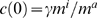 that is not relevant for the steady state distribution. Numerical simulations show that the signal propagation is restricted to smaller and smaller values of the activation ratio 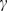 as the parameters 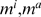 are reduced. This is shown in Fig. 8. Note, that even in the case when the saturation constants are the same, *m^i^* = *m^a^*, the threshold for signal propagation is smaller than one.

## Discussion

Cascades of protein modification cycles form the backbone of many signaling pathways, such as MAPK and GTPase cascades, which integrate signals from numerous plasma-membrane receptors and transmit information to distant cellular targets, including the nucleus [4],[14],[37]. A hallmark of these signaling cascades is the spatial separation of activation and deactivation processes to different cellular compartments [19],[32]. In such spatially distributed cascades, the first signal transducer can be activated at the cell membrane by a membrane-bound enzyme, e.g., a kinase or a guanine nucleotide exchange factor for GTPase cascades, and deactivated in the cytosol by an opposing enzyme, e.g., a phosphatase or a GTPase activating protein [18]. If a subtle balance between the rates of activating and deactivating enzymes, e.g., kinases and phosphatases, is not properly maintained, the phosphorylated kinase concentrations can drop even in close proximity to the activation membrane source, and the phosphorylation signals decay before reaching the targets.

The results of the present paper have identified the general conditions for the robust signal propagation and determined when the activation signal stalls near the membrane for an arbitrary number of consecutive layers in a cascade. The signals that spread through a cascade generate a set of stationary activation profiles. When the ratio of the deactivation and activation rates, *γ* , is small, these activation profiles have almost identical plateau levels for successive kinases and shifted in the space relative to each other by a roughly constant distance towards the center of the cell. We expressed analytically the amplitude and the width of successive activation profiles that spread the signals into the cell interior and examined the effect of saturation of the reaction rates on the propagation of the phosphorylation signals. This precipitous descend in the signals at different distances from the plasma membrane provides digital, switch-like localization cues that are more structured and robust than the information carried by a concentration gradient that emerges in a single (de)phosphorylation cycle [38].

Importantly, we found that the control of the spatial signal propagation is dramatically different from the control of transient temporal responses for spatially homogenous cascades [17],[39]. Whereas the persistence of transient activation in the spatially uniform cascade depends mainly on the phosphatase activities [17], our results show that the spatial spread of activation from the membrane into the cell is determined by the ratio of the kinase and phosphatase activities and by their degree of saturation. Likewise, the maximal amplitude of propagating activation profiles depends on the activity ratios of phosphatases and kinases and not mainly on the kinases, as the amplitude of the temporal responses for spatially homogenous cascades [17].

More complex spatial patterns of active kinases are generated when the ratios of phosphatase and kinase activities are different along the cascade (see Fig. S6). The spatial structure of activity gradients also strongly depends on the size and shape of the cell [24]. An interesting consequence of the activity profiles produced by spatially distributed cascades is that at different cascade levels, the kinase activities at the nuclear membrane change with the cell size. As the cell grows, the distance between the cell membrane and nucleus increases, and consequentely the activity of proteins at the boundary is turned off one by one for increasing cascade levels. This suggests a signaling mechanism that conveys information about the cell size to the nucleus. This mechanism may play a role in the control of cell division cycle. The step structure of the concentration profiles ensures that a sharp change in concentration takes place when the cell reaches a certain size, representing a robust digital switch-like signal.

In this work we have not considered the effects of feedback and feedforward loops, which may lead to more complex spatial structures and temporal dynamics [19]. In fact, it has recently been shown that bistability in protein phosphorylation cascades generates phosphoprotein waves that propagate from the surface deep into the cell interior [33]. For the cascade levels localized to the cytoplasm, if a downstream kinase stimulates the activation of the upstream kinase (directly or via a regulatory circuit in the cytoplasm), a resulting bistable switch generates a trigger wave that propagates with nearly constant amplitude and velocity [33]. However, although such a wave relays the signal over increasingly long distances, it also destroys the positional information delivered by the successive activation profiles for monostable cascades, which we analyzed here.

It is instructive to compare spatially distributed reaction cascades to other reaction-diffusion systems exploited in mathematical models of biological phenomena. Traveling front or pulse solutions in reactions with multiple steady states or with excitable dynamics (e.g., the Hodgkin-Huxley model) produce concentration distributions that propagate in space with a constant speed, but rarely generate heterogeneous spatial structures at steady states [40]. On the other hand, the classical mechanism of morphogenesis based on the Turing instability leads to the formation of stationary concentration patterns driven by different diffusivities of the reacting species [41]. Although often this condition is not satisfied for biological systems, the Turing mechanism has been suggested to explain the formation of skin pigmentation patterns [42], hair follicle distribution [43] and other biological patterns. Because of their translational symmetry, periodic Turing patterns are not always suitable for providing positional information. The concentration distribution generated by a spatially distributed reaction cascade can provide a simple and robust spatial pattern, in which the distance from the source (e.g., cell membrane) is encoded into the local concentrations. There is an important distinction between the Turing patterns and patterns originated in spatially extended protein cascades considered here. Heterogeneous Turing patterns arise spontaneously due to diffusion driven instability and symmetry breaking, transforming an initially homogeneous spatial distribution [41],[44]. The spatial patterns considered here involve the initial non-homogeneity of the media, which is brought about by the spatial separation of the opposing activator and deactivator enzymes that localize to different cellular structures, namely the membrane and cytoplasm [45]. This type of reaction-diffusion mechanism may also play a role at larger scales in the development of multi-cellular systems where positional information and growth guide cell proliferation and differentiation events.

## Supporting Information

Text S1 Exact solutions for small concentrations.
(0.05 MB DOC)

Figure S1 Temporal evolution of the active form concentration profiles for the sixth level, c6(x), obtained by numerical integration of Eq. (8) with γ = 0.1 and initial conditions for different values of ν at times: (A) t = 5; (B) t = 10; (C) t = 50; (D) t = 10^4^.
(1.30 MB EPS)

Figure S2 Temporal evolution of the active form concentration profiles c6(x) obtained by numerical integration of Eq. (8) with γ = 0.1 and ν = 1 for different initial conditions at times: (A) t = 1; (B) t = 10; (C) t = 170; (D) t = 10^4^.
(1.33 MB EPS)

Figure S3 Active form concentration profiles, cn(x), obtained numerically from Eq. (8) with γ = 10 at different times: (A) t = 0.01; (B) t = 0.1; (C) t = 1; (D) 10^4^.
(1.30 MB EPS)

Figure S4 (A) Stationary total concentration as a function of n for small values of γ. The dashed lines are the linear fit to the data. (B) The slope p as a function of γ obtained by linear fit to the data. The dashed line represents p = 1.3(1−γ)ln(1/γ). The inset in B shows the same figure but with the x-axis in the logarithmic scale.
(1.15 MB EPS)

Figure S5 Temporal evolution of the active form concentration profiles c6(x) obtained by numerical integration of Eq. (13) with γ = 0.1, ν = 1, ma = 1 and mi = 1 for different initial conditions at times: (A) t = 1; (B) t = 10; (C) t = 170; (D) t = 10^4^.
(1.46 MB EPS)

Figure S6 Stationary concentration profiles, cn(x), obtained by numerical integration of Eq. (5) far from saturation with cn(x,t = 0) = 0, D = 1, kn^a^ = 1 for all n, k4^i^ = 10, and kn^i^ = 0.1 for n = 1,2,3,5,6.
(0.28 MB EPS)
