# Supplementary material for: Positional Information Generated by Spatially Distributed Signaling Cascades
Source: PLoS Comput Biol. 2009 Mar 20;5(3):e1000330. doi: 10.1371/journal.pcbi.1000330 (PMC2654021; doi:10.1371/journal.pcbi.1000330)
Supplement: Text S1 — Exact solutions for small concentrations. (0.05 MB DOC) [file pcbi.1000330.s001.doc]

**Text S1**

## Exact solutions for small concentrations

It can be shown that Eq. (9) has a solution of the form $c_{n}=Q_{n}^{(n-1)}(x)e^{-x}$ where $Q_{n}^{(n-1)}$ is a polynomial of order $n-1$ corresponding to the $n$th-level. If we write $Q_{n}^{(n-1)}$ as $Q_{n}^{(n-1)}(x)=\int_{m=0}^{n-1} q_{n}^{(m)}x^{m}$, where $q_{n}^{(m)}$ is the coefficient of the *m*th order term in the solution corresponding to the *n*th level of the cascade, then the relation between the coefficients is given by

$\left\{\begin{matrix} 2\gamma(n-1)q_{n}^{(n-1)}=q_{n-1}^{(n-2)}; & & \\ & & \\ 2\gamma(m+1)q_{n}^{(m+1)}-\gamma(m+1)(m+2)q_{n}^{(m+2)}=q_{n-1}^{(m)}, & m=0,1,...,n-3. & . \end{matrix}\right.$ (A1)

In the case $\gamma$>1 the approximation remains valid near the left boundary and using the boundary condition there we obtain an additional equation for the zero order coefficient in the polynomial at the different $n$-levels, $q_{n}^{(0)}$, which reads $q_{n}^{(0)}=q_{n}^{(1)}$. For $\gamma$<1 the approximation is not valid near the boundary *x*=0 and the coefficients $q_{n}^{(0)}$cannot be exactly calculated.

We can use (A1) to obtain successively an explicit solution for the different levels to Eq. (9). Considering$c_{1}(x)=c_{1}(0)e^{-x}$, for the next five levels we have

$\begin{matrix} c_{2}=\frac{c_{1}(0)}{2\gamma}(x+1)e^{-x}, \\ c_{3}=\frac{c_{1}(0)}{(2\gamma)^{2}}(\frac{1}{2}x^{2}+\frac{3}{2}x+\frac{3}{2})e^{-x}, \\ c_{4}=\frac{c_{1}(0)}{(2\gamma)^{3}}(\frac{1}{3!}x^{3}+x^{2}+\frac{5}{2}x+\frac{5}{2})e^{-x}, \\ c_{5}=\frac{c_{1}(0)}{(2\gamma)^{4}}(\frac{1}{4!}x^{4}+\frac{5}{12}x^{3}+\frac{15}{8}x^{2}+\frac{35}{8}x+\frac{35}{8})e^{-x}, \\ c_{6}=\frac{c_{1}(0)}{(2\gamma)^{5}}(\frac{1}{5!}x^{5}+\frac{1}{8}x^{4}+\frac{7}{8}x^{3}+\frac{7}{2}x^{2}+\frac{63}{8}x+\frac{63}{8})e^{-x}. \end{matrix}$ (A2)
